# Progress toward national estimates of police use of force

**DOI:** 10.1371/journal.pone.0192932

**Published:** 2018-02-15

**Authors:** Joel H. Garner, Matthew J. Hickman, Ronald W. Malega, Christopher D. Maxwell

**Affiliations:** 1 Department of Criminology and Criminal Justice, Portland State University, Portland, OR, United States of America; 2 Department of Criminal Justice, Seattle University, Seattle, WA, United States of America; 3 Department of Geography, Geology and Planning, Missouri State University, Springfield, MO, United States of America; 4 School of Criminal Justice, Michigan State University, East Lansing, MI, United States of America; London School of Economics, UNITED KINGDOM

## Abstract

This research builds on three decades of effort to produce national estimates of the amount and rate of force used by law enforcement officers in the United States. Prior efforts to produce national estimates have suffered from poor and inconsistent measurements of force, small and unrepresentative samples, low survey and/or item response rates, and disparate reporting of rates of force. The present study employs data from a nationally representative survey of state and local law enforcement agencies that has a high survey response rate as well as a relatively high rate of reporting uses of force. Using data on arrests for violent offenses and the number of sworn officers to impute missing data on uses of force, we estimate a total of 337,590 use of physical force incidents among State and local law enforcement agencies during 2012 with a 95 percent confidence interval of +/- 10,470 incidents or +/- 3.1 percent. This article reports the extent to which the number and rate of force incidents vary by the type and size of law enforcement agencies. Our findings demonstrate the willingness of a large proportion of law enforcement agencies to voluntarily report the amount of force used by their officers and the relative strengths and weaknesses of the Law Enforcement Management and Administrative Statistics (LEMAS) program to produce nationally representative information about police behavior.

## Introduction

The authority to use physical force is one of the most distinguishing and controversial aspects of American policing. While use of force has been a topic of both public and scholarly interest for many years, this interest intensified in the wake of the 2014 deaths of Eric Garner and Michael Brown, and several subsequent controversial fatal police actions. In addition to public protests and a kind of ‘crisis of confidence’ in the police, these events also put the spotlight a long standing problem: the lack of national data about police use of force. When individuals ranging from members of the public to members of Congress asked, “How often does this happen?” the disappointing answer they received was, “We don’t know.” Later that same year, President Obama created a Task Force on 21st Century Policing whose members were charged with examining such issues as how to strengthen public trust and police legitimacy. One of many recommendations of the President’s Task Force was that police department use of force policies should require the collection and reporting of data on all officer-involved shootings to the Federal government. Separately, the Federal Bureau of Investigation (FBI) has since taken some initial steps toward collecting data on fatal uses of force from State and local agencies.

There is an extensive body of research about the amount of force used and the characteristics of the police, the residents, the incidents and the community that are associated with more or less force. For detailed reviews of this literature, see [1–3]. However, most of this research is based on either a single or a small number of jurisdictions or parts of jurisdictions. Another difficulty is that the data sources that are employed to measure force vary widely; studies use individual police reports, surveys of law enforcement agencies, systematic observations of police public contacts, interviews with residents or suspects, surveys of the general population and compilations of media accounts [2]. Our understanding of the amount of force used by American police is limited further because of the lack of a consistent definition or measurement of force from jurisdiction to jurisdiction, from study to study, and over time. For instance, some studies are limited to police shootings [4], deaths resulting from police shootings [5] or incidents where various types of weapons are used [6]. At the other extreme are studies that count shouting, threats of arrest, and other types of language as a use of force [7] and some studies of force using systematic field observations include hundreds of use of force incidents, none of which involve the actual use of a weapon by a police officer [8].

Use of force measurement is also complicated by variation in the units of analysis. Studies can report the number of incidents where one or more types of force are used; the number of types of force used in particular incidents, the number of officers that use force in a particular incident, or some combination of these three. Understanding the amount of force is complicated further by diverse computations of the *rate* of force. Numerous studies have computed rates of force by dividing the number of force incidents by either the size of the resident population [9], the number of sworn officers [10], arrests [11], police pubic contacts [7], potentially violent encounters [12], or calls for service [13].

Efforts to understand the impact of these diverse methods and measures are hindered by the fact that no study uses more than one source of data, one measure of force, or one rate of force; therefore it is difficult to try to calibrate across studies the impact of different data sources, measures or rate computations. Under these conditions, it is not surprising there is a great deal of variety in the available reports with regard to what constitutes force and exactly how much force is used by American police agencies. Among 36 studies recently reviewed [2], the smallest average rate of force reported– 0.1 percent of calls for service [14]–is three hundred times smaller than the highest reported rate of force: 30.0 percent of suspect encounters [15]. The wide range of reported rates of force suggests that there are wildly different understandings of what does and does not constitute “force” and that there is a substantial amount of imprecision in how force is measured and rates of force are computed.

### National estimates of police use of force

For more than 30 years, criminologists have regularly complained about to the absence of comprehensive, accurate, and timely national-level data on police use of lethal force [1,16,17], with one going so far as to lament that journalists did a better job reporting such events than criminologists or the Federal government [18]. The national controversy over the number of Black residents killed by the police find little agreement between protestors [19] and high level law enforcement officials [20] except for the need for accurate and up-to-date national data on the number of homicides by the police. However, even if current efforts using open sources [21,22] or planned efforts for surveying law enforcement agencies [23] were to be successful, those efforts alone will tell us virtually nothing about the nature and extent of the far larger (and largely unknown) amount of force used by police officers which do not result in deaths or even serious injury. As the widely publicized incident in Baltimore involving Freddy Gray demonstrated [24], the difference between incidents of lethal and non-lethal force can easily reflect the behavior of medical transport services and the proximity of high quality trauma centers and not necessarily the behavior of residents or sworn police officers [25,26].

At the present time, there are two sources of data that have been used to produce national estimates of the amount and rate of force used by the police in the United States. The first source is the Police Public Contact Survey (PPCS) conducted every three years between 2002 and 2011 by the Bureau of Justice Statistics (BJS) as a supplement to the National Crime Victimization Survey (NCVS), a nationally representative sample of households [7,27–29]. The second type of data on uses of force comes from government funded but privately implemented sample surveys of law enforcement organizations [9,10,14,30]. While surveys of law enforcement organizations can capture all types of force used regardless of whether the force used results in injury or death, by the nature of its design, the PPCS cannot capture force incidents that result in death. Both of these approaches—the surveys of residents and surveys of law enforcement agencies—have methodological strengths and weaknesses; however, because of design limitations and implementation difficulties, neither of these approaches has yet produced reliable national estimates of the amount of force, the rate of force, or the correlates of force.

### Police public contact survey

For each of the four waves of the PPCS, the intended sample was all English-speaking persons over 15 years of age that responded to the NCVS. Designed and funded by BJS, the NCVS is implemented on a continuing basis by the U.S. Bureau of the Census. After responding to questions about their crime victimizations during the past 6 months as part of the NCVS, a sub-sample of individuals are asked to complete a supplemental interview about their face-to-face contacts with the police during the past 12 months. Among those individuals reporting face to face contact with the police, the PPCS asks, among other things, if the police used force, what type of force, and whether the respondent was arrested.

While the design of the PPCS program is to measure how often the public has contact with the police, the actual implementation of this survey varied from the design in several ways. For instance, in 2011, the NCVS survey was completed by 62,280 (88.0 percent) of the intended nationally-representative sample of 70,773 individuals (the NCVS is a household survey; the figures used here rely on BJS counts of individuals) but the 2011 national estimates of force were produced based on responses from 41,408 individuals; this is 66.5 percent of the NCVS respondents and 58.5 percent of the originally selected nationally representative sample (Table 1). BJS reports that 18 percent of the intended NCVS sample were excluded because individuals did not speak English, refused to complete the survey, were non-interviews, or were included in the NCVS only by proxy—another person in their family reported their victimization experiences. In addition, for methodological purposes, the 2011 design of the PPCS called for 15 percent of the available sample to use the 2008 survey instrument [7]. In all four waves of the PPCS, BJS publications used the relationship between the use of force and the age, race and sex of the respondents in the survey to estimate the amount of force experienced by the intended respondents who were not surveyed and then used the sampling probabilities of the entire survey to produce a national estimate for the amount and rate of force. Thus, in 2011, the responses from 58.5 percent of the nationally representative NCVS sample were used to produce national level estimates of force.

**Table 1:** Police-public contact survey (PPCS) intended and actual interviews, 2011.

	Number of Persons	Percent of intended NCVS sample	Percent of actual NCVS sample
NCVS intended sample older than 15 years	70,773	100.0	-
NCVS non-respondents	8,493	12.0	-
NCVS respondents	62,280	88.0	100.0
No interviews, refused, or non-English speaking	10,907	15.4	17.5
Proxy interviews	2,127	3.0	3.4
Total persons excluded	13,034	18.4	20.9
PPCS sample, 2011	49,246	69.6	79.1
PPCS questionnaire, 2008	7,838	11.1	12.6
PPCS questionnaire, 2011	41,408	58.5	66.5

In another set of exclusions to the PPCS sample, a number of other individuals whose residences were eligible to be included in the NCVS during the time period covered by the PPCS but were in hospitals, mental institutions, halfway houses, jails or prisons at the time the PPCS survey was conducted were not included NCVS or PPCS samples. The actual size of all these exclusions are unknown and are not incorporated into PPCS estimates of force (however, it has been estimated that including recently jailed inmates would increase the PPCS estimates of force by 17 percent [2]). Thus, the PPCS’s sample may not well represent the population of all residents who come into contact with the police, have force used against them or are arrested, including individuals living in circumstances which make them more likely to have such interactions with the police than individuals of a similar age, race or sex.

In addition to the survey response rates, a second limitation of the PPCS is the many and varied ways that it has measured police public contacts and uses of force over time. For instance, the four BJS published reports from the PPCS have each reported force differently. Four types of force were reported for the 2002 PPCS–pushed, kicked, pointed a gun and other. For the 2005 PPCS, the use of a chemical agent was reported. For the 2008 PPCS, the use of electrical weapons and shouting/cursing were added. Lastly, for the 2011 PPCS, handcuffing was added as a use of force. For none of the PPCS waves is the discharge of a firearm reported. Beyond using a variety of force types over the years, the PPCS also changed the format of the force question. In the first three waves, the PPCS first determined if the respondent had a face to face contact with a police officer in the past year. If they did, the respondents were then asked a series of questions about the nature of the contact, including whether the police used force against them. If the respondent says “yes” to the initial use of force question, they were then asked about what type of force was used. In 2011, the PPCS survey was changed in two major ways. First, each respondent was asked the same general question about experiencing force used in prior surveys as well as nine specific questions about particular types of force. The second change involved asking questions about force only of respondents who were 1) stopped by the police on the street or 2) stopped while driving a car.

Based on these methods, the PPCS reported that force was used or threatened against less than one third of one percent of residents in 2002 and 2005. In 2008, the rate dropped to about one-fourth of one percent (Table 2). Based on these rates, the estimated number of uses of force was 664,280 for 2002, 707,522 for 2005 and 574,070 for 2008. The reports from the first three waves of the PPCS also distinguish between three types of behavior: 1) physical force, 2) verbal threats, and 3) shouting and cursing. Physical force, described as any physical contact including pushing, hitting, kicking, and weapon use, constituted about 55 percent of these three behaviors. Thus, the rates of physical force for those years are about half of what is reported for all types of force—less than one sixth of one percent of U.S. residents—which is among the lowest rates of force reported in existing studies of police use of force [2].

**Table 2:** Police-public contact survey (PPCS) contacts, arrests, use of force, 2002–2011.

	2002	2005	2008	2011
U.S. residents older than 15 years	215,536,800	228,040,120	236,511,830	241,404,142
**Incidents**				
Police public contacts	45,278,900	43,537,370	40,015,000	62,936,500
Arrests	1,302,417	N.R.	N.R.	N.R.
Uses of force	664,280	707,520	574,070	N.R.
Physical force	332,115	389,136	341,728	N.R.
Verbal Force	503,500	266,028	219,042	N.R.
Threats of force	N.R.	194,568	145,094	N.R.
Shouting or cursing	N.R.	71,460	73,948	N.R.
**Drivers**				
Drivers	192,687,190	202,539,650	209,218,860	212,298,850
Stopped	16,783,500	17,825,140	17,663,000	26,404,200
Uses of force	188,822	142,919	160,000	1,610,656
Arrests	448,094	427,803	459,238	264,042
**Street stops**				
Street stops	N.R.	N.R.	N.R.	1,433,300
Uses of force	N.R.	N.R.	N.R.	364,058
**Rates**				
Contacts per 100 residents	21.01	19.09	16.92	26.07
Arrests per resident	0.01	N.R.	N.R.	N.R.
Force per 100 residents	0.31	0.31	0.24	N.R.
Physical force per 100 residents	0.73	0.17	0.14	N.R.
Force per 100 arrests	51.00	N.R.	N.R.	N.R.
Physical force per 100 arrests	25.50	N.R.	N.R.	N.R.
Traffic stop per 100 drivers	8.71	8.80	8.44	12.44
Force per 100 drivers	0.10	0.07	0.08	0.76
Arrest per 100 drivers	0.23	0.21	0.22	0.12
Force per 100 arrested drivers	42.14	33.41	34.84	610.00
Street stop per 100 residents	N.R.	N.R.	N.R.	0.6
Force per 100 street stops	N.R.	N.R.	N.R.	25.4

N.R. = Not Reported

The BJS reports for the 2002, 2005 and 2008 PPCS provide the rate and number of uses of force separately for drivers in traffic stops. For all drivers, the estimated number of incidents of any type of force in those years was 188,822, 142,919, and 160,000 and the rates of any type of force for drivers were less than one tenth of one percent. The 2011 PPCS produced an estimate of 1,610,565 incidents, a count ten times larger than the average number of incidents from the prior three waves of the PPCS. This difference is probably the result of new screening questions about contacts with the police and about uses of force. Drivers in a traffic stop are the only group for which a measure of force is reported in both the 2008 and the 2011 reports and the reported rates of force per driver increased almost 10 times from 0.08 percent [29] to 0.76 percent in 2011 [7].

There are two additional problems with using the PPCS to measure force. First, the triennial BJS reports [7,27–29] only use the most recent incident where a contact with the police involved force; additional contacts where a use of force is reported are not counted. The second problem not addressed by the 2011 revisions is the inconsistency in the PPCS and other national estimates of arrests, traffic stops and motor vehicle accidents. In the 2002, 2005 and 2008 PPCS surveys, the estimated number of drivers arrested ranges from 427,803 to 459,238 (See Table 2). In the 2011 survey, the PPCS estimated that there were only 264,042 drivers arrested [7]. In addition, during 2002 –the only year that estimates of arrests for all residents are reported in the PPCS–the estimated number of arrests for all residents was 1.3 million, which is about 10 percent of the FBI national estimates of 12 million arrests during 2011 [31].

Using the PPCS to estimate uses of force is problematic due to sampling issues, question revisions, and the inconsistent definition and measurement of force and arrest used across the various versions of the survey. The exclusion of non-English speakers, jailed offenders, and other persons not covered in the NCVS with proxy interviews skews the representativeness of the PPCS sample in ways not addressed by the statistical weights. The measurement of police public contacts and subsequent arrests vary substantially across waves of the PPCS further limiting use of the four waves of the PPCS to produce rates of force per police contact or per arrest. Lastly, just as victimization surveys cannot measure homicide, the PPCS cannot measure police use of lethal force.

### Measuring force with administrative surveys

An alternative approach used to measuring police use of force is to survey law enforcement agencies. There are four independent research efforts that have attempted to capture the existing information in law enforcement records to estimate the amount and rate of force using surveys of law enforcement agencies in the U.S. [9,10,14,30]. While all of these studies surveyed State and local general purpose law enforcement agencies, they varied greatly in the size and nature of their sample, the rate at which agencies responded to the survey and the rate at which responding agencies responded to questions about the amount of force (Table 3). These four surveys also varied in how force and rates of force were defined and measured.

**Table 3:** National surveys of law enforcement agencies about police use of force.

	Pate & Fridell (1993)	Alpert & MacDonald (2001)	IACP (2001)	Taylor, et al. (2010)
Years Studied	1991	1996	2000	2005–2008
Agency types included	State & local police & sheriffs	Local police & sheriffs with > 50K population	Any law enforcement agency	State & local police & sheriffs
Population of agencies	15,801	N.R.	N.R.	16,072
Sample size	1,697	832	No sampling	950
Survey respondents	1,111	571	228	518
Agencies reporting force	529	265	228	327
Percent reporting force	31.2	31.9	N.A.	34.4
Weapon availability	Yes	N.R.	No	Yes
Required reporting	Yes	N.R.	No	Yes
Unit of analysis	Incidents	Incidents	Incidents	Incidents / reports
Types of force measured	18	3	5	13
Reported use of force	N.R.	76	3.51	23
Rate of force denominator	Per sworn officer	Per 100,000 population	Per 10,000 calls for service	Per agency by force type

N.R. = Not Reported

In 1992, the Police Foundation surveyed U.S. state and local law enforcement agencies to collect information about their use of force policies and practices [10,32]. A sample of 1,697 state and local police agencies and sheriff’s offices were asked if they mandated reporting for 18 different types of police behavior ranging from firearm discharges to handcuffing. Among the 1,111 responding agencies, all state agencies and about 95 percent of sheriffs and local police agencies mandated reporting the number of individuals shot and shot at. Mandatory reporting for incidents that involved the use of other weapons ranged from 93.8 percent for firearm discharges to 70.2 percent for chemical agents; the rate of mandated reporting varied from 66.6 percent to 19.2 percent for incidents where the police made physical contact without using any weapons [10]. Pate and Fridell reported the rate of force per 1,000 sworn officers for each of the 18 types of force, separately for sheriffs, county police agencies, city police agencies and State agencies. For instance, based on the 557 responding municipal police departments, Pate and Fridell reported there were 4.1 firearm discharges for every 1,000 sworn officers; among the 409 city agencies that reported the use of weaponless tactics, there were 272 uses of weaponless tactics for every 1,000 sworn officers. Thus, they report rates per force type per officer for 18 different but overlapping samples of agencies. In addition, a force incident could involve more than one type of force. Given these limitations, Pate and Fridell did not report rates of force for all types of agencies nor did they summarize the amount or rate of force across all 18 types of police behavior.

In 1997, 571 agencies responded to a survey sent by the Police Executive Research Forum (PERF) [9] to a total of 832 sheriff’s departments and municipal police agencies in U.S. jurisdictions over 50,000 in population. Among the 571 responding agencies, 265 of them provided information on the number of times their officers used physical force, a chemical agent or any other weapon to control a suspect during 1996. For the 265 agencies with data on force, the data were combined on all three types of force with a resulting median rate of force equal to 76 incidents per 100,000 residents. It was also reported that across agencies their rate of force ranged from .24 to 868 incidents per 100,000 residents [9]. In a multivariate model, it was reported that there were lower rates of force in Northeastern states and in jurisdictions with lower violent crime rates, and no differences in the rates of force for agencies that were accredited or agencies that had employee unions [9].

In 2001, the International Association of Chiefs of Police (IACP) presented the final report of a project that measured five types of police uses of force–physical, chemical, electronic, impact and firearm–over a seven year period from 1994 through 2000 [14]. This project relied on the voluntary submission of reports to the IACP from U.S. law enforcement agencies. Over the life of the project, there were a total of 564 annual submissions including 177,000 incidents of force, of which more than 80 percent involved the use of weaponless tactics. There were 112 agencies participating in 1995 and 228 in 2000. In the other five years, fewer than 100 agencies contributed data on incidents of force. Based on data from 1999 (the last year for which complete data were available) the IACP reported that police used force at a rate of 3.61 times per 10,000 calls for service to police agencies.

In 2005, researchers associated with PERF [30] developed a survey that asked questions about each agency’s use of 10 types of police behavior during 2003, 2004 and 2005, and sent it to a nationally representative sample of 950 law enforcement agencies. A total of 516 agencies responded to the 2005 survey. In 2009, another survey was sent to the 516 agencies asking about the same 10 items; 327 of the 516 agencies provided information about their uses of force in 2006, 2007 and 2008. The data were used to report the average rate of force per agency for each of the ten types of force for 2005 through 2008. For instance, the lowest rate of force was for the number of civilians shot and killed per law enforcement agency, which was 0.05 in 2005, 2007 and 2008. In 2006, the rate for this type of force was 0.06. On the other hand, the most frequently reported type of force–empty hand tactics–ranged from 18.56 per agency in 2005 to less than 12.00 for the other three years. Similar to [9,10], the responses were weighted to conform to the characteristics of their original sample, but they did not report an estimate for the amount or rate of all uses of force for the United States.

These four agency surveys are fairly consistent in the types of police behavior that they include in their measure of force (S1 Table); however, two of them provide a more detailed listing [10,30]. Virtually all of the types of police behavior in all four studies involve the use of physical force; the one exception is pointing or unholstering a firearm [10,30]. There is less agreement among these four studies on whether force is measured by force type, by incident or by officer report. In Pate & Fridell’s study [10] the unit of measurement is the type of force not the force incident. Taylor, et al. [30] explicitly addressed this issue by requesting that the agencies specify whether their counts of force are based on incidents or on separate reports from all the officers on the scene. They found that most agencies can report force counts by incident or by officer reports; 35 percent of the agencies could only report incidents and 13 percent could only report officer counts. The remaining 52 percent could produce both, and so they reported separate analyses using incident counts and officer counts [30]. Alpert and MacDonald [9] and IACP [14] did not specify which units of measurement were used but they combined counts across types of force, which suggests they had incident level data.

### Assessment of prior research

The four surveys of law enforcement organizations have limitations similar to those with the PPCS. First, the survey response rates ranged from about one half to two thirds of their original sample and only about one third of their original samples provided information on uses of force. The PPCS and three agency surveys using national probability samples used sampling weights to account for survey and item nonresponses. Unlike the PPCS, the three agency surveys did not report standard errors or confidence intervals for the estimates they produced. The three studies with national samples used data from one third of their sample to estimate the other two thirds of their sample. Perhaps the biggest difficulty in making sense out of these four studies is their use of diverse computations for a rate of force. Pate and Fridell [10] report rates separately for each type of force but all of them are based on the number of sworn officers. Alpert and MacDonald [9] compute a rated based on the population of the jurisdiction served. The IACP report [14] uses calls for service, and Taylor, et al. [30] compute a rate based on the number of force incidents per agency. Even if these studies had measured uses of force in exactly the same manner, the use of alternative rate computations makes comparisons across studies difficult.

These eight publications–four from the PPCS and four from agency surveys–represent the best available efforts at producing national estimates of police uses of force in the United States. However, their low response rates, their diverse definitions and measurements of force and their individualized approaches to constructing a rate of force limit their usefulness. This research will address those limitations by using data on uses of force from a survey of law enforcement organizations that has a relatively high response rate and is based on common policies requiring the documentation of force items. In addition, we consider alternative approaches to imputing missing data and the construction of rates of force.

The one consistent finding across every prior study of police use of force is that force of any type is a rare phenomenon. Regardless of the samples, measures, or analyses used, uses of force are rare among all residents, among all police public contacts and occur in only a small proportion of incidents where residents are arrested. Since there are about 250 million adults in the U.S. and, in a single year, only 0.24 percent to 0.31 percent of them report uses of force, threats of force or shouting and cursing, the PPCS and other surveys of the general public require samples larger than 50,000 respondents, making it an inefficient approach to obtaining reliable estimates of force. While there are some important benefits to using resident surveys to study force, the overriding characteristic of American policing is that most sworn officers work in a small number of large departments. For instance, in 2008 almost half of the 765,000 sworn officers in the U.S. worked in the 409 agencies with 250 or more sworn officers. Three quarters of the sworn officers worked in the 2,500 agencies with 50 or more sworn officers [33]. With approximately 15,000 state and local law enforcement agencies in the U.S., a sample of about 3,000 agencies is sufficient to produce estimates of police behavior with small confidence intervals [34].

Much of what is known about policing in the U.S. is derived from establishment surveys of law enforcement organizations. This research approach originated with the Kansas City Police Department as a mechanism to understand the pay and benefits provided by major U.S. law enforcement agencies [35,36]. Perhaps the most well-known and used survey of American law enforcement organizations is the Uniform Crime Reporting program developed by the International Association of Chiefs of Police and, since 1931, operated under the auspices of the FBI as part of a collaborative effort by state and local law enforcement agencies to use consistent definitions and measurements over time and between agencies to report the nature and amount of crime and arrests for crime in the United States [37]. In separate organizational surveys, the FBI also reports the number of agencies and officers [38], the number of assaults on law enforcement officers [38], and detailed characteristics of homicides [39]. In addition, there have been numerous surveys of law enforcement organizations conducted by academic researchers and by national law enforcement professional organizations, such as PERF, the Police Foundation, The National Sheriff’s Association, and the IACP [40].

## Design of this research

The Bureau of Justice Statistics initiated the Law Enforcement Management and Administrative Statistics (LEMAS) program in 1987. Based on nationally representative samples of all State and local general purpose law enforcement agencies in the U.S. and implemented nine times between 1987 and 2013, the LEMAS surveys are the primary source of descriptive data about American policing [41,42]. The survey response rates for the LEMAS program have ranged from 95.2 percent in 1987 to 91.7 percent in 2007. These response rates are unparalleled in research on American law enforcement organizations, even though the LEMAS program includes a large number of small agencies, which most other surveys of law enforcement organizations do not even attempt to include. Past LEMAS surveys have produced national level estimates of the organizational size, employee demographics, educational requirements, budgetary resources, community policing activities, administrative policies, available technology as well as the characteristics of a variety of formal policies of state and local law enforcement agencies in the U.S. Since 1989, BJS has archived LEMAS data for public use at the University of Michigan and, except for the NCVS, these data are the source for more independent criminological research than any other BJS program [43].

Despite the strengths of the LEMAS program, a National Academy of Sciences review of BJS programs criticized several aspects of the LEMAS program, primarily for its narrow emphasis on administrative data, for the absence of links to crime and other data collected by the FBI, for its lack of timeliness, and its emphasis on the administrative characteristics of law enforcement organizations, such as police personnel, equipment and formal polices [44]. The Academy Panel argued that future LEMAS surveys should do more to measure police behavior and performance. It recommended that the LEMAS program adopt a shorter survey instrument, with less time between survey waves, using a core of consistently asked questions along with one or more sets of supplemental questions addressing topical issues in a manner similar to the relationship of the PPCS to the NCVS.

### Methods to estimate use of force

Even though the “core and supplement” design had never been tested in surveys of police organizations and the willingness of police agencies to report sensitive data about police behavior in the LEMAS survey was unknown, the 2013 LEMAS survey instrument included core questions about the number and types of personnel and supplemental questions about community policing, information systems, officer safety and police use of force [34]. The final sample design called for a total of 3,336 agencies selected from within seven sampling strata based on three agency types—local police departments, sheriff offices, and primary state police agencies—and six categories of agency size based on the number of sworn officers—100 or more, 99 to 50, 40 to 25, 24 to 10, 9 to 5, 4 to 2, and 1. All agencies employing 100 or more full time sworn officers were sampled with certainty and this included all 50 primary state police agencies. Agencies were selected with decreasing probability as the size of the agency declined and these probabilities were slightly different for police departments and sheriff officers [45]. This design creates 15 sampling strata–seven size categories each for local police departments and sheriff offices and one more for all state police. The 2013 survey obtained responses from 2,826 agencies for an overall response rate of 88 percent [45]. In a manner similar to prior LEMAS surveys, the 2013 LEMAS survey asked agencies which of seven types of weapons, five types of weaponless tactics, and two types of threats of force were authorized for use by some or all sworn personnel. For each of the 14 types of behavior, the 2013 LEMAS survey also asked about the agency policies for documenting the use of these weapons, tactics and threats. The 2013 LEMAS survey asked each agency for both the total number of use of force *incidents* and the total number of *reports* of force from individual officers for 2012 in a manner similar to Taylor, et al.’s [30] approach. The 2013 LEMAS program only asked about the total amount of force used during 2012 and did not ask agencies to count and report a number for each of the 17 types of police behavior.

In 2015, BJS released two reports about local police departments based on the 2013 LEMAS survey; neither report provided any information about uses of force [46,47]. Shortly thereafter, the Department of Justice in general and the BJS LEMAS program in particular were criticized by a New York Times article for collecting use of force data that the Times asserted was “almost useless” and having nothing to contribute to then current nationwide controversies about police use of force [48]. In addition to the issue of counting force separately by incidents and by reports, the Times authors asserted that there was no uniformity in which types of force agencies document and that a number of departments, including large departments in Baltimore, Houston, and New York City, did not report information about their uses of force in the LEMAS survey. The article quoted a prominent police researcher as saying that the situation was “a national embarrassment.” The expert also asserted that law enforcement agencies would not provide use of force data unless the Federal government provided an incentive or made it a requirement for Federal grant money.

### Documenting police behavior

As previously noted, the 2013 LEMAS survey asked agencies which of 14 types of police behavior were authorized by their department. In addition, if the force type was authorized, did departmental polices require documentation of their use. Seven of these behaviors involved the uses of weapons and four involved weaponless tactics. The survey also asked about the use of severe restraints and two threats of force—the display of a firearm or the display of an electrical weapon. Based on the 2013 LEMAS data file [45], we computed the percentage of agencies that require documentation of each of the 14 types of police behavior. These descriptive statistics show consistently high rates across all types of weapons and weaponless tactics (Table 4). For instance, the 2013 survey showed that documentation of firearm use is required by 96.4 percent of agencies. In 2012, more than 90 percent of agencies also require that all other types of weapon use be documented. In addition, the survey results show that the generally less severe but much more frequently used weaponless tactics are documented by more than 87 percent of law enforcement agencies. Thus, contrary to Apuzzo and Cohen’s [48] *assumptions* about the LEMAS survey, the actual data demonstrate consistently high rates of documenting the use of weapons and weaponless tactics among American law enforcement agencies; in addition, those rates have increased substantially since Pate and Fridell’s survey.

**Table 4:** Police behavior documented.

		LEMAS (2013): Percent Documented if Authorized	Pate & Fridell (1991): Percent Mandated Reporting
Weapon Used:			
	Handgun	96	95
	Baton	93	82
	Impact weapons	94	60
	Electrical weapon	92	48
	OC spray/foam	94	N.A.
	Other chemical agent	91	N.A.
	Any chemical agent	N.A.	72
	Soft weapon / bean bag	94	N.A.
Tactics Used:			
	Closed hand	89	N.A.
	Open hand	87	N.A.
	Closed or open hand	N.A.	66
	Take down	87	30
	Neck hold	93	66
	Severe restraint	78	N.A.
Weapons Threatened:			
	Display handgun	54	33
	Display electrical weapon	49	N.A.

N.A. = Not Applicable

### Counting incidents and officer reports of force

Appuzzo and Cohen’s critique [48] of the LEMAS data appears uninformed about the purpose and potential value of collecting data on force incidents as well counts force reported by individual officers. Contrary to their general assertions, among the 2,826 agencies in the 2013 LEMAS survey, 1,508 (53.4 percent) reported an estimate of the number of force incidents in 2012; in addition, 1,119 agencies (39.6 percent) reported a count based on officer reports (Table 5). We supplemented the data reported in LEMAS with some open-source information on use of force incidents, drawn from agency annual reports, data reported on agency websites, Commission on Accreditation of Law Enforcement Agencies (CALEA) reports, and one newspaper investigation using police records. These efforts resulted in data on uses of force in 2012 from an additional 32 agencies—29 with incident data and 3 with report data. This supplemental search focused on the largest agencies with missing data on uses of force in the LEMAS survey. Adding these 32 agencies resulted in incident data from 1,537 and report data from 1,122 agencies. We used these additional data because they represented that agencies were willing to report uses of force to the public—which critics asserted they would not do—even if they did not, in this instance, report those estimates to the LEMAS survey.

**Table 5:** Steps to estimate incidents of force for LEMAS, 2013 sample.

		Number of Agencies	Percent of Agencies
Total agencies in sample		2,826	100.0
Force measures:			
	Force incidents measure	1,537	54.4
	Officer reports measure	1,122	39.7
	Force incidents and officer reports measured	529	18.7
	Only officer reports of force	589	20.8
	Force estimated from incident and report data	2,100	74.3
	No data on force incidents	726	25.7
Arrest measures:			
	Reports arrests to UCR program	2,253	79.7
	Reports arrests for violence to UCR program	2,200	77.8
	Data on arrests but not force	567	20.1
	Force estimated from force and arrest data	2,667	94.4
Measures of sworn officers:			
	Reports of sworn officers in LEMAS, 2013	2,826	100.0
	Force estimated from number of sworn officers	161	5.6
	Composite measure of force from four sources	2,826	100.0

Importantly, these two groups of agencies (reporting force incidents; reporting officer reports) are not mutually exclusive; 529 (18.7 percent) agencies reported both incident counts and officer reports. Within this group of agencies, we estimated the average ratio of officer reports to incidents. After removing values of zero and greater than one, the average ratio of incidents to reports was found to be equal to 0.64. This ratio implies that the average incident involved about one-and-a-half officers (i.e., 1/.64). Since there was very little variance in this estimate across agency size, type, and sampling strata, we used this overall ratio to estimate the number of force incidents for the 567 agencies that reported the number of officer reports of force but not incidents of force. For example, if an agency reported 500 officer reports of force, we would estimate those 500 reports represented (500*.64) = 320 force incidents.

These steps produced a total of 2,100 agencies with incident-level data, or 74.3 percent of all sampled agencies, a substantial improvement over prior research in which the largest number of agencies reporting force was 526. In addition, the 74.3 percent of agencies reporting an estimate of uses of force is more than twice the percent of agencies reporting a measure of force in any prior survey of research organizations (See Table 3). Furthermore, the 2,100 agencies reporting data on uses of force represent 62.9 percent of the intended LEMAS sample of 3,336 agencies, which is comparable to the 2011 PPCS where the respondents comprised 58 percent of the intended nationally representative sample.

Among the agencies that responded to the 2013 LEMAS survey, almost 75 percent provided data on their agency’s use of force. While this is an improvement over prior organizational surveys about police uses of force, these data are not missing at random. For instance, 86 percent of the 50 primary state police agencies and 81 percent of 574 local police agencies with 100 or more officers provided a force estimate; on the other hand, the force response rate was 67 percent for all 717 sheriff offices and for the 229 police agencies and sheriff offices with fewer than 5 sworn officers. In their criticism of the 2013 LEMAS program, Apuzzo and Cohen [48] cite experts–but no research–to assert that American law enforcement agencies will not report how much force they use unless they are coerced or provided incentives. Our examination of the 2013 LEMAS data demonstrates that when asked, in 2012, a large proportion of American law enforcement agencies did, in fact, either voluntarily report uses of force in the LEMAS survey or had made efforts to make the number of force incidents public through other means. It is worth noting that this level of self-reporting was achieved at the same time that the Civil Rights Division of the U.S. Department of Justice was more frequently than ever bringing suits against law enforcement agencies for their potentially illegal or racially discriminatory use of force policies or practices [49].

### Linking LEMAS with FBI arrest and employment data

One of the recommendations of the National Academy of Sciences report on BJS programs was that the LEMAS survey data should include the FBI alphanumeric identification code (ORI) for law enforcement agencies so that LEMAS agency characteristics can easily be linked for analysis purposes to data about offenses, arrests and employees that are collected and reported by the FBI [31, 38]. However, the 2013 LEMAS data did not include the agency identification code from the BJS 2008 Census of State and Local Law Enforcement Agencies [50], which served as the population of agencies from which the 2013 LEMAS sample was drawn. In addition, the 2013 LEMAS data initially released in July 2015 did not include the agency ORI codes. Because of the lack of common identifiers, variation in agency names, and errors in various agency descriptive codes, there was no simple method to link records in the 2013 LEMAS, 2008 BJS Census, and the 2013 FBI data on arrests.

Using a combination of automated sorting and personal examination, it was possible to link records from these three data files. The initial step was to link records in the 2013 LEMAS and the 2008 BJS Census files based on the state and agency names. LEMAS agencies that did not match were sorted by state and agency type and name in both files and compared visually. Potential matches were checked using data on state, city, postal zip codes and agency size and type. The matching was complicated by the existence of many small agencies with similar names. In some states, there are multiple agencies with exactly the same name but operating in different counties. For instance, in New Jersey, there are three agencies named Franklin Township Police Department. The two in Somerset and Gloucester are in the 2013 LEMAS; the one in Hunterdon County is not.

Once the LEMAS agencies were matched to the corresponding agencies in the BJS Census file, this process used the census identification code to identify the corresponding agencies in the 2013 BJS Crosswalk file [51]. The crosswalk file included FBI ORI codes for all but 75 of the LEMAS agencies. As part of our effort to extract arrest and employment data from FBI files, we identified ORI codes for 73 of these 75 LEMAS agencies.

After completing our initial analyses, an examination of subsequent releases of the 2013 LEMAS data revealed a heretofore unannounced addition of ORI codes for all but two agencies. The ORI codes provided by BJS matched our codes in all but 18 (0.6%) of the 2,826 agencies. Comparing these unmatched agencies with FBI records determined that our initial match was accurate in 12 out of these 18 instances. As a result, the ORI codes for six LEMAS agencies were revised; FBI arrest records were available for three of these six agencies.

Matching the arrest data with the primary state law enforcement agencies required special attention. The principle state agency in the LEMAS 2013 file matched arrest records for only 15 states. However, the FBI also records arrest reports by using ORI codes for sub-state agencies—typically state police barracks. In California and Rhode Island, both recording methods are used. Special attention and programming was needed to identify those records and to aggregate them up to the record of the primary state agency. The SPSS programs used to identify the FBI arrest records and to match those records to agencies in the 2013 LEMAS are included as part of the data and documentation for this project that are archived at OpenICPSR (http://doi.org/10.3886/E101132V1).

One of the first things that we learned from matching these three files is that among the 2,826 respondents to the 2013 LEMAS program, 2,253 (79.7 percent) agencies reported their total arrests and 2,200 (77.8 percent) agencies reported the number of arrests for a violent offense to the FBI for 2012 (Table 5). The 75.2 percent response rate for force in the LEMAS survey is fairly comparable to the 79.5 percent response rate for arrests in the FBI survey even though this was the first time BJS had asked law enforcement agencies about uses of force and the FBI has been collecting arrest data for more than 50 years.

### Addressing the problem of missing data about force

Prior research using probability samples of agencies to measure police use of force have addressed the differential response rates among agencies either by ignoring it or by weighting the responding agencies by their type and size to correspond to their frequency among all State and local agencies [9,10,30]. The later approach implicitly assumes that the use of force behavior of officers in agencies that did not respond is similar to the behavior of officers in agencies that did respond.

We chose a different approach to address the problem of missing data that is similar to the approach employed by BJS in the PPCS [7,27–29]. In these reports, BJS used the relationship between amount of force used and the age, race and sex of PPCS respondents to estimate the amount of force used against non-respondents. Our initial effort used the relationship between the amount of force reported and the number of arrests for violent offenses to estimate the number of uses of force. We used this arrest measure because it captures the extent to which law enforcement agencies are engaged with violent offenders. Among the agencies having both use of force data and arrest data in the integrated BJS–FBI file, use of force is highly correlated with arrests for violent offenses (Pearson’s *r* is equal to .78).

Of the 2,100 agencies that reported uses of force and the 2,200 agencies that reported data on arrests for violent offenses to the FBI, 1,646 of those agencies reported both. Using data from those 1,646 agencies, we estimated bi-variate regression equations that predicted the amount of force based on the number of arrests for violent offenses. To address the observed variation within sampling strata, we produced separate bi-variate models within each agency type and size strata. Because only four local police agencies and two sheriff’s offices with one sworn officer had missing data on force, we combined those strata with the next largest agency size within their agency type. In addition, all state police agencies had 100 or more officers so we combined them with the strata with the local police agencies with 100 or more officers. Of the 726 agencies with missing data on the amount of force used in the 2013 LEMAS sample, 565 reported data on arrests for violent offenses to the FBI. Using our regression equations based on the type and size of the agency, we produced estimates of force for each of those 565 agencies.

As a result of the use of force measures in the LEMAS survey and this estimation procedure, we produced estimates of force for 2,665 agencies, leaving only 161 (5.6 percent) of the agencies with missing data on uses of force. We addressed this last missing data issue by using median imputation methods originally recommended for the LEMAS program by the Census Bureau [52]. We computed the ratio of force incidents to the number of sworn officers among the 95 percent of the LEMAS sample for which we had force estimates. This ratio varies substantially between agency type and agency size, so, for each sampling strata, we computed the median of the force to sworn officer ratio. We identified 27 agencies whose force estimates were extremely high (greater than 4) and replaced those values with the median amount of force within their sampling strata. We then estimated the amount of force for the last 134 agencies by multiplying the number of sworn officers in each agency by the strata specific force to officer ratio. The final step in our estimation procedure is to apply the 2013 LEMAS sampling weights for each stratum. These are the same sampling weights employed by BJS that account for the original probability-based sampling design and the survey response rate for the 2013 LEMAS survey [46,47].

## Findings

Table 6 displays our findings about the amount of force used by State and local law enforcement agencies in the U.S. during 2012. Based on the procedures we have set out above, we estimate that during 2012 State and local law enforcement agencies in the U.S. used force in 337,594 incidents; our probability based national estimate has a standard error of 5,342 incidents or 1.6 percent. Thus, the 95 percent confidence interval for our national estimate ranges from 327,124 to 348,064 incidents. (We used the SPSS Complex Samples Module [53] to implement the Taylor linearization method to estimate variances for this study). Standard errors for each stratum are provided in S2 Table. Table 6 also displays the extent to which the amount of force varied by the type and size of law enforcement agencies. Three-quarters of all force incidents were reported by local police agencies and nearly 60 percent of the incidents were reported by the 1,009 police and sheriff agencies with 100 or more officers, confirming once again the highly concentrated nature of American policing in a relative small number of large agencies.

**Table 6:** Use of force incidents by agency type and size, 2012.

	Local police departments		Sheriff offices		Primary state agencies		All agencies	
Number of sworn officers	Number of agencies	Force estimate	Number of agencies	Force estimate	Number of agencies	Force estimate	Number of agencies	Force estimate
All sizes	12,326	253,361	3,012	76,295	50	7,938	15,388	337,594
100 +	659	139,657	350	50,258	50	7,938	1,059	197,852
50 to 99	800	33,731	322	10,478	-	-	1,122	44,209
25 to 49	1,542	33,742	578	6,338	-	-	2,120	40,080
10 to 24	2,842	27,708	907	5,948	-	-	3,749	33,657
5 to 9	2,507	10,800	565	2,546	-	-	3,072	13,347
2 to 4	2,630	5,660	260	643	-	-	2,890	6,302
1	1,346	2,064	30	84	-	-	1,376	2,148

Because differences in the amount of force used could reflect the number of residents in those jurisdictions, in Table 7 (panel ‘A’) we produce a rate of force per 10,000 residents. While there is great similarity in these rates across strata, the rates of force per resident are higher in police departments and sheriff offices with fewer than 10 sworn officers. This per resident rate also produces lower rates for sheriff offices compared to local police but that could reflect the fact that sheriff offices are frequently not the primary patrolling agency in their own county. Similarly, the primary state police agencies generally focus on traffic enforcement and do not regularly respond to calls for service. Implicit in Table 7 (panel ‘A’) is the hypothesis that the amount force used is generated by the number of people served by the law enforcement agency.

**Table 7:** Rates of force per 10,000 residents, per 100 officers, and per 100 arrests for violent offenses, by agency type and size.

Number of sworn officers	(A) Incidents per 10,000 residents (n = 15,388)			
	Local police departments	Sheriff offices	Primary state agencies	All agencies
All sizes	11.0	2.8	0.3	10.8
100 or more	10.4	2.9	0.3	-
50 to 99	10.9	2.9	-	-
25 to 49	11.5	2.0	-	-
10 to 24	12.1	2.4	-	-
5 to 9	13.9	4.0	-	-
2 to 4	12.1	4.1	-	-
1	15.9	5.7	-	-
Number of sworn officers	(B) Incidents per 100 sworn officers (n = 15,388)			
	Local police departments	Sheriff offices	Primary state agencies	All agencies
All sizes	51.6	39.1	13.6	45.4
100 or more	46.3	40.8	13.6	41.0
50 to 99	63.1	44.1	-	57.3
25 to 49	61.5	27.2	-	51.3
10 to 24	59.2	32.6	-	51.7
5 to 9	53.4	48.8	-	52.4
2 to 4	49.6	53.5	-	50.0
1	82.1	90.3	-	82.4
Number of sworn officers	(C) Incidents per 100 arrests for violent offenses (n = 7,631)			
	Local police departments	Sheriff offices	Primary state agencies	All agencies
All sizes	18.7	25.2	14.6	19.6
100 or more	16.8	29.5	14.6	18.3
50 to 99	18.9	19.2	-	18.9
25 to 49	22.6	15.0	-	21.3
10 to 24	25.1	20.2	-	24.3
5 to 9	26.8	41.0	-	28.9
2 to 4	39.0	49.4	-	39.9
1	22.9	127.3	-	26.0

Note: Panel ‘C’ is limited to agencies that reported both force and arrests.

Table 7 (panel ‘B’) reports the rate of force per 100 sworn officers by the type and size of law enforcement agencies. Implicit in this table is the idea that the amount of force is determined in great part by the number of sworn officers available to use force. However, these findings show that the number of incidents is not a direct result of the number of officers and that, in fact, the ratio of force to officers varies by agency type and is generally lower for larger agencies and higher for smaller agencies. Compared to the national average of 45.4 uses of force per 100 sworn officers, the lowest rates of force are associated with state police agencies (13.6) and sheriff offices (39.1). The average rate for local police agencies with 100 or more officers at 46.3 is close to the national average but all other size categories for local police exceed the national average with the highest overall rate of force per sworn officers belong to the local police agencies (82.1) and small sheriff offices (90.3) with only 1 officer. The lowest rate of force per 100 sworn officers is for the primary state police agencies.

We recommend caution in interpreting the findings in Table 7. Two error prone interpretations of Table 7 are that 1) local police agencies use more force than sheriff offices or state police agencies and 2) small law enforcement agencies use more force than large agencies. The major basis for this error is assuming that the size and type of agencies are the main or only determinate of the amount of force used.

Because our estimates are based on a systematic sample of law enforcement agencies, we are able to produce confidence intervals around our estimates to account for sampling error and to use those confidence intervals to calculate whether differences between agencies of different types and sizes are statistically significant. These tests provide a basis for assessing whether the different estimates could stem from sampling errors, but they do not constitute causal analyses about the relationship between size and type of agencies and the amount of force. Moreover, multivariate statistical analyses of these data might provide a more nuanced description of the distribution of force but they would not necessarily provide a rigorous test of the causal forces involved in producing more or less incidents of force. Multivariate analyses assist in generating some theoretically-relevant hypotheses about those causal relationships but rigorous tests of those hypotheses require more than descriptive statistics. For example, Ridgeway [54] uses an analytical design to assess risk factors in police uses of lethal force among Philadelphia police officers. Systematically produced descriptive statistics, like those presented here, can provide a baseline measure of police behavior but are limited in their ability to support causal analyses [55].

In Table 7 (panel ‘C’), we present what we hope will be an additional constraint on any causal misinterpretations of these descriptive findings. Table 7 (panel ‘C’) shows that the average rate of force per 100 arrests for a violent offense is 19.6. This rate also varies by the type and size of the reporting agencies. Implicit in this measure is the hypothesis that officers use force based in great part on the nature of the work they do and that more arrests for violent offenses means more contact with potentially violent offenders and, therefore, a greater likelihood to use force. Controlling for the number of arrests for violent offenses still produces different average rates between agency types and sizes. Table 7 (panel ‘C’) shows that sheriffs’ offices have a higher rate of force per 100 arrests at 25.2 than local police agencies at 18.7, which is just the opposite ranking of agency types from Table 7 (panel ‘B’). Thus, depending upon which rate is selected–per sworn officer, per resident, or per arrest for violent offenses–the relationship between uses of force and the type and size of an agency varies considerably. There is variability in the amount and rates of force by agency size and type but the descriptive data in this report is not a sound basis for asserting that the size and type of agencies causes that variability.

## Discussion

This research examines the utility of the 2013 LEMAS survey for producing national estimates of the amount and rate of use of force used by State and local law enforcement agencies. Compared to prior efforts to use surveys of law enforcement organizations to measure force, this effort has achieved substantial improvements in survey response rates, the number and proportion of agencies providing data on force, and in addressing the problem of missing data. These improvements demonstrate that the LEMAS program can be used successfully to capture data on police behavior such as the number of force incidents, and future LEMAS surveys should build on the progress made in 2013.

Despite widespread assertions to the contrary, three quarters of all surveyed state and local law enforcement agencies employing 85 percent of the sworn officers in the U.S. voluntarily reported their use of force in the 2013 LEMAS survey. Therefore, the responsibility for the lack of national data on police use of force cannot easily be attributed to the unwillingness of State and local law enforcement community to report; rather, the lack of future progress toward national statistics on police uses of force now lies more clearly on the police use of force research community, the national law enforcement professional associations, and professional statisticians at federal agencies responsible for collecting data on police behavior.

The main uncertainty about these estimates is the quality and consistency of the reported data on force. The 2013 LEMAS survey demonstrated that virtually all law enforcement agencies in the U.S. have formal policies requiring documentation for the use of all weapons and weaponless tactics. If such policies did not exist, it would be difficult to expect that agencies could report how much force they used in the prior year. However, it is not known how consistently those policies are implemented or the extent to which agencies surveyed compiled accurate records of the number of those incidents. Moreover, these are agency self-reported data, consisting of aggregated officer self-reported data. Like all self-reported data about behavior, these reports are likely to represent some degree of under counting. Although the amount of under counting is unknown, our use of self-reports is likely to produce estimates lower than the actual amount of force. As such, our estimates should be regarded as conservative.

In addition, there are no national standards defining each type of force similar to the national standards established by the FBI’s Uniform Crime Reports defining types of crime, and the 2013 LEMAS survey did not specify precisely which police behaviors were and were not to be counted as force. Some agencies may have included threats of force or the use of restraints in their responses to the LEMAS survey, while others did not. This type of measurement problem is not unique to the issue of force, to this wave of the LEMAS survey, to other BJS survey programs, to all studies of police behavior or to social research in general. In its first three waves, the PPCS used a similarly general question about use of force. Given the consistent policies about documenting weapon use and weaponless tactics, future LEMAS surveys should be able to explicitly define which of these police behaviors should and should not be included in agency estimates of force incidents.

This research does not necessarily provide definitive national estimates of the amount or rate of force used by the police (and we regard them as conservative estimates) but it does demonstrate that considerable progress has been made toward making such an estimate possible, plausible and accurate. Until future research can improve on the 2013 LEMAS response rates and develop and implement a consistent measure of force, our estimates appear to be improvements over prior efforts to survey law enforcement organizations on this issue and at least as methodologically strong as estimates produced from the PPCS.

### Measuring force with LEMAS and PPCS

Given the production of LEMAS-generated estimates of the amount and rate of force used by state and local law enforcement agencies, it seems appropriate to compare and contrast BJS’s two approaches to this task. The LEMAS program surveys organizations; the PPCS survey interviews individuals. Both programs use probability sampling and weight the responses they receive to produce estimates that are nationally representative of law enforcement agencies and residents, respectively. The PPCS program is dependent on the consistency and accuracy of individual perceptions and memory of contacts with the police reported to the Census Bureau; the LEMAS program relies on the consistency and accuracy of reports from law enforcement organizations reported to BJS. At the present time, neither program has consistently used a clear definition of what types of police behavior are and are not incidents of force.

There are real differences between what the PPCS and the LEMAS survey can do with their measures of force. The PPCS can compare the rates of force between different types of people but it cannot consider the nature of law enforcement agency or the jurisdiction in which the force incident occurred. The LEMAS program can contribute nothing about the person against whom force is used but can incorporate the characteristics of the law enforcement agency and the jurisdiction where the police officer works. Neither program can easily collect data about the characteristics of the law enforcement officer involved in each incident. The PPCS has and the LEMAS could measure changes in the amount and rate of force over time. Both the PPCS and the LEMAS programs would benefit from a classification system that determines which types of police behavior do and do not constitute force, and if that behavior does constitute force, what type of force it is. It is unlikely that the BJS survey programs could accomplish this on their own. What is needed is a collaborative effort among law enforcement agencies and statistical experts to produce reportable, meaningful, reliable, and consistent measures of different types of force.

It may be possible to leverage the current efforts by the FBI to collect data on fatal police shootings toward this broader goal. The FBI effort is a deliberate multi-phase undertaking, involving agency stakeholders very early in the design phases, and with subsequent phases contingent on the results and lessons-learned from their initial forays into collecting the data [23]. As this data collection platform develops, it may be possible to expand from the narrow focus on lethal force into very specific types of force (e.g., the use of conducted energy devices, such as the Taser, or the use of impact weapons, such as a baton), and later into other types of physical force, while encouraging agency conformity to reporting practices. A similar expansion of scope has occurred over the history of the development of the UCR program. With time, and with a continued deliberate strategy (including the resources to support research and development, as well as auditing), the FBI could become a primary source of national data on police use of force, and these efforts could help to inform the PPCS and LEMAS programs.

There appear to be strengths and weaknesses in both approaches and, in the future, these programs may function as two complementary approaches measuring force similar to the way that the NCVS and the UCR are currently seen as two complementary but distinct ways to measure crime [56]. Both BJS programs that measure police use of force are not anywhere as fully developed as the NCVS or the UCR programs but the prospects for making additional progress in both programs seems promising. While it could be heartening that the national estimate of 337,594 incidents of force produced by the 2013 LEMAS program is very similar to the 2008 PPCS estimate 341,728 incidents of physical force, it should also be noted that, in 2011, the PPCS changed the format of its use of force survey questions and BJS reported a much larger estimate of 1,610,656 incidents of any type of force just among people stopped by the police in their car [7]. Differences of this magnitude are not likely the result of changes in police behavior nor are they likely to stem from differences in the sampling approaches of the PPCS and the LEMAS program.

The lack of a common definition for and measurement of different types of force is likely to be a large contributor to these differences and to the differences reported in the prior research literature of police use of force [2]. Without a common understanding of which police actions are and are not uses of force, the progress made by the PPCS and the LEMAS programs is unlikely to lead to the production of regular, comprehensive and reliable national estimates of how much force is used by American law enforcement officers. The amount of missing data is the second major problem for both the PPCS and the 2013 LEMAS. For purposes of comparison with existing research, this paper used the same general approach to imputation used by the PPCS and the LEMAS program to produce national level descriptive statistics [52]. Some independent scholars have used these methods when conducting multivariate analyses of use of force data from the PPCS [2,57]. However, this approach does not explicitly consider whether the data are missing at random. Little and Rubin [58] argue that imputation of missing values for dependent variables is essential for getting unbiased estimates of regression coefficients. Thus, while alternative approaches to missing data are not likely to substantively change our national estimates of force, future efforts to collect and analyze national estimates of police use of force will benefit from reducing the amount of missing data and being more attentive to the relative strengths and weaknesses of alternative approaches to imputation of the data that are missing [59,60].

Linking the LEMAS data to the FBI arrest data was a crucial element in being able to reasonably impute missing data on uses of force. If this integrated BJS-FBI file was also linked with jurisdiction specific demographic data from the U.S. Census Bureau, the prospects for better understanding the correlates of force would be greatly enhanced. Including census data would not eliminate the limitations of the 2013 LEMAS use of force data, but it would provide a solid basis to make progress on testing hypotheses about the agency and jurisdictional characteristics associated with the amount of force used by the police.

This research documents the progress that has been accomplished in estimating the amount of force used by the police in the United States. This progress is exemplified by the use of a survey with an 86% response rate and for which 73% of the responding agencies provided an estimate of force. These response rates far exceed research using agency surveys and even exceed the response rate for the Police Public Contact Survey. In addition, our findings demonstrate that the policies of large proportions of law enforcement agencies already call for documenting the use of weapons and tactics. By merging the LEMAS survey with FBI data on arrests, this study revealed the strong correlation between reports of force and reports of arrests for violent arrests. While this research used that correlation to impute missing data, that correlation is also of theoretical importance in demonstrating a strong relationship between legitimate police behavior and the use of force. Lastly, this research reported rates of force based on the population of the jurisdiction, the number of sworn personnel and the number of arrests for violent offenses, which revealed the diverse distribution of these rates among the type and size of law enforcement agencies.

The progress reported here, while substantial, is not sufficient to produce reliable national estimates of force. The data from the 2013 LEMAS survey cannot assess whether law enforcement agencies actually document force consistently, that agencies define force in a similar manner, or that agencies will be truthful in reporting their uses of force to the public. These three issues are currently being addressed by a joint BJS-FBI effort and the results of that effort may provide the additional improvements needed to produce a better understanding of the amount and correlates of force in the United States.

## Supporting information

S1 Table Types of force used in organizational studies of force. (DOCX)

S2 Table Standard errors for Table 6. (DOCX)
