# Supplementary material for: Progress toward national estimates of police use of force
Source: PLoS One. 2018 Feb 15;13(2):e0192932. doi: 10.1371/journal.pone.0192932 (PMC5813980; doi:10.1371/journal.pone.0192932)
Supplement: S1 Table — (DOCX) [file pone.0192932.s001.docx]

| Pate & Fridell (1993) | Alpert & MacDonald (2001) | IACP (2001) | Taylor, et al. (2010) |
| --- | --- | --- | --- |
| Weapon use | | | |
| Shot and killed | Use of a weapon | Firearm | Shot and killed |
| Wounded not killed |  |  | Wounded not killed |
| Shot but not hit |  |  | Shot but not hit |
| Electrical devices |  | Electronic | CED |
| Batons |  | Impact | Batons |
| Other impact devices |  |  | Other impact devices |
| Flashlight |  |  | Flashlight |
| Chemical agents | Chemical agent | Chemical | Chemical agents |
| Dog attacks or bites | N.R. | N.R. | Canine bites |
| Vehicle ramming |  |  | Vehicle ramming |
| Weaponless tactics | | | |
| Twist lock/wrist lock | Physical force | Physical | Empty hand tactics |
| Bodily force |  |  |  |
| Swarm |  |  |  |
| Firm grip |  |  |  |
| Come-alongs |  |  |  |
| Handcuff/leg restraint |  |  |  |
| Neck restraints |  |  | Neck restraint |
| Threats and other types of force | | | |
| Unholstering weapon | N.R. | Other | Pointing weapon |

**Supporting Information Table 1. Types of Force Used In Organizational Studies of Force**

N.R.= Not Reported
