# Supplementary material for: Progress toward national estimates of police use of force
Source: PLoS One. 2018 Feb 15;13(2):e0192932. doi: 10.1371/journal.pone.0192932 (PMC5813980; doi:10.1371/journal.pone.0192932)
Supplement: S2 Table — (DOCX) [file pone.0192932.s002.docx]

**Supporting Information Table 2. Standard Errors for Table 6**

| Number of sworn officers | Local police departments | Sheriff offices | Primary state agencies | All agencies |
| --- | --- | --- | --- | --- |
| All sizes | 4,604 | 2,708 | 0 | 5,342 |
| 100 or more | 3,705 | 2,440 | 0 | 4,436 |
| 50 to 99 | 1,497 | 1,028 | - | 1,816 |
| 25 to 49 | 1,708 | 395 | - | 1,753 |
| 10 to 24 | 1,283 | 290 | - | 1,315 |
| 5 to 9 | 548 | 269 | - | 611 |
| 2 to 4 | 471 | 92 | - | 480 |
| 1 | 386 | 29 | - | 388 |
